# On the art of stealing chloroplasts

**DOI:** 10.7554/eLife.64057

**Published:** 2020-11-20

**Authors:** Paulo Cartaxana, Sónia Cruz

**Affiliations:** 1 Department of Biology, University of AveiroAveiroPortugal; 2 Centre for Environmental and Marine StudiesAveiroPortugal

**Keywords:** *Acetabularia acetabulum*, biophysics, Elysia timida, green algae, kleptoplasty, photosynthetic sea slugs, Other

## Abstract

Sea slugs increase the longevity of the chloroplasts they steal from algae by limiting the harmful side-effects of photosynthesis.

**Related research article** Havurinne V, Tyystjärvi E. 2020. Photosynthetic sea slugs induce protective changes to the light reactions of the chloroplasts they steal from algae. *eLife* **9**:e57389. doi: 10.7554/eLife.57389

It is a common perception that animal cells do not have chloroplasts, the organelles that performs photosynthesis. Yet, a small number of sea slugs are able to incorporate carbon from CO_2_ by performing photosynthesis using sequestered chloroplasts from the macroalgae in their diet. These sea slugs use the stolen chloroplasts (also known as kleptoplasts) to produce metabolites, such as sugars and fatty acids, which can then be transported from the slug’s digestive gland to other tissues in the body (Cruz et al., 2020).

During photosynthesis, photons of light are absorbed by complexes of pigments and proteins called photosystems, which reside in the membranes of thylakoids (the compartments in chloroplasts where light-dependent reactions occur). This energy is then transferred to chlorophyll molecules called P680 and P700, which form part of photosystem II and photosystem I, respectively.

The energy absorbed by photosystem II is used to transfer electrons from P680 to p700 via a series of carrier molecules in the photosynthetic electron transport chain. P680 regains the lost electrons through the splitting of water molecules. As the electrons are passed through the chain they lose energy, which is used to translocate protons into the thylakoids. This creates a difference in the concentration of protons inside and outside the thylakoids, which the chloroplasts use to drive the production of ATP (Figure 1). The energy absorbed by photosystem I is used to transfer electrons via several intermediates to the enzyme ferredoxin-NADP reductase. This enzyme catalyzes the production of NADPH by donating electrons to NADP^+^, which can form a bond with a proton. ATP and NADPH are energy-containing molecules that are used subsequently in the light-independent reactions of photosynthesis (the Calvin cycle) and other cell processes.

**Figure 1.**
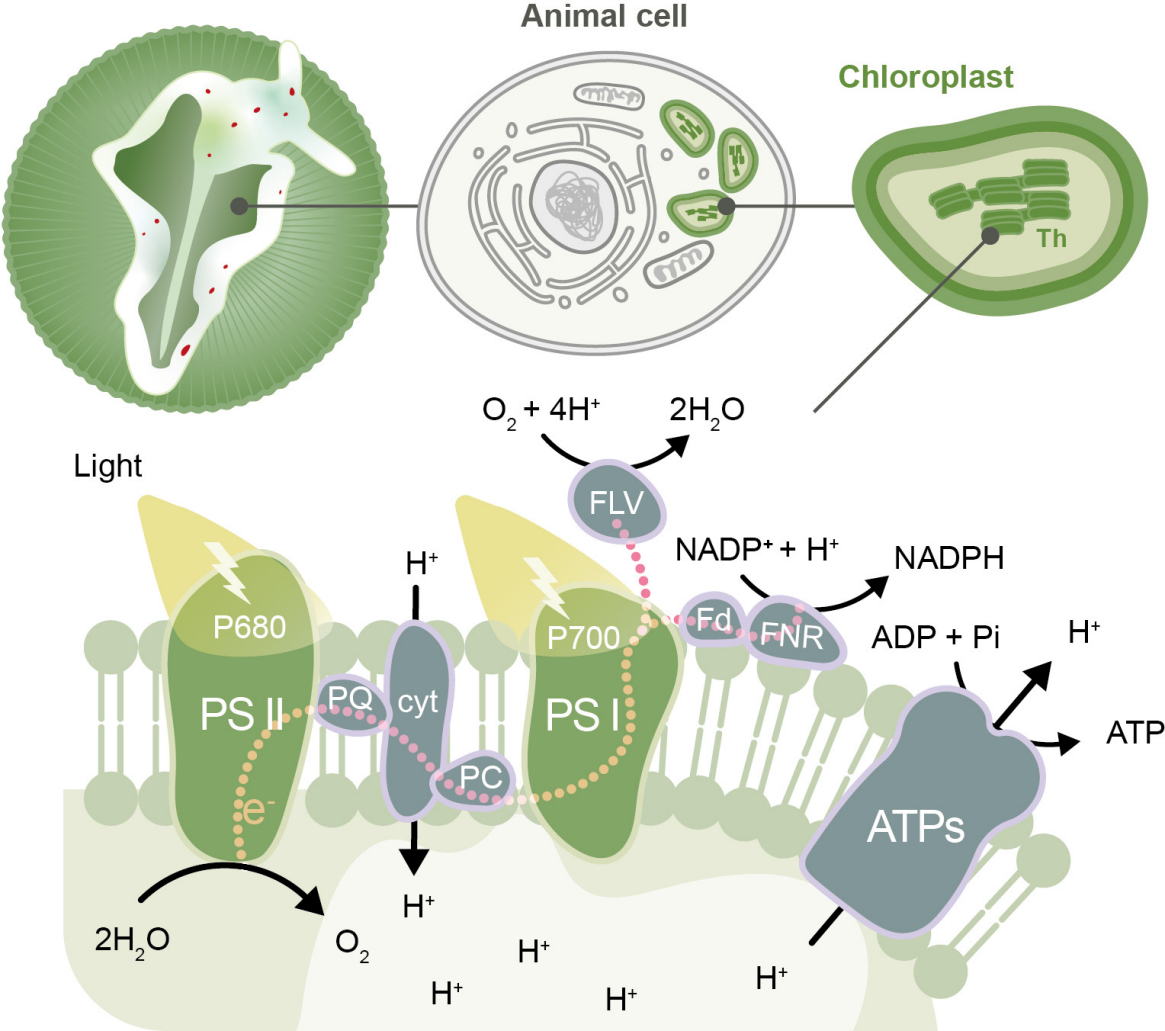
The photosynthetic electron transport chain in the kleptoplasts of the sea slug *Elysia timida.* Top: schematic representation of the sea slug *E. timida* (left), showing the cells (center) that contain the functional chloroplasts (right) stolen from its food source *Acetabularia acetabulum*. Bottom: schematic of the photosynthetic transfer chain in the thylakoid (Th) membrane of kleptoplasts in *E. timida* sea slugs. From the left: the P680 chlorophyll in photosystem II (PSII) absorbs light and transfers an electron to plastoquinone (PQ), which, through a series of steps, passes the electron to the P700 chlorophyll in photosystem I (PSI). P680 regains its electron from the splitting of two molecules of water (H_2_O) into four protons (H^+^) and one oxygen (O_2_) molecule. Photosystem I absorbs light and transfers an electron to ferredoxin (Fd) and its accompanying enzyme (ferredoxin-NADP reductase, FNR), which uses the electrons to convert NADP^+^ to NADPH. This sequence of reactions creates a build-up of protons inside the thylakoids that can be transported out into the stroma of the chloroplasts by an enzyme called ATP synthase (ATPs), releasing the energy needed to make ATP, a molecule that fuels chemical reactions in cells. If the photosystems become overloaded with photons, this leads to the production of molecules that can harm the sea slug’s cells. Kleptoplasts are protected from this damage by maintaining plastoquinone oxidized in the dark (so it is able to accept more electrons when photosystem II absorbs light), and by donating the excess electrons from PSI to alternative molecules, such as flavodiiron proteins (FLV). The other molecules shown in the figure are cytochrome *b*_6_/*f* (cyt), plastocyanin (PC), adenosine diphosphate (ADP) and inorganic phosphate (Pi).

The metabolites generated through photosynthesis play an important role in sea slug survival and fitness in periods of food scarcity (Cartaxana et al., 2017). Nevertheless, the wide variety of compounds produced by the biochemical reactions in the chloroplasts can pose risks to animal cells. The most obvious threat for these ‘solar-powered’ sea slugs are reactive oxygen species (ROS), which are formed by the addition of electrons to oxygen: these reactive molecules can severely damage tissues and eventually cause cell death (Dorrell and Howe, 2012). Complex compounds that block oxidation (the loss of electrons) and mechanisms for dissipating excessive energy have both been proposed to play a protective role in kleptoplast-bearing sea slugs (Cartaxana et al., 2019; Torres et al., 2020). However, despite over 50 years of research, it is still unclear how kleptoplasts are maintained in animal cells.

Now, in eLife, Vesa Havurinne and Esa Tyystjärvi from the University of Turku, report a detailed analysis of the photosynthetic light reactions occurring in the kleptoplasts of the sea slug *Elysia timida* (Havurinne and Tyystjärvi, 2020). Havurinne and Tyystjärvi maintained populations of over 500 sea slugs and their food source, the green macroalga *Acetabularia acetabulum* in the laboratory. The algae and sea slugs were then exposed to different light, O_2_ and CO_2_ levels, and various chlorophyll fluorescence techniques were used to measure light energy absorption and photosynthetic electron transfer in the thylakoid membranes of the chloroplasts.

Havurinne and Tyystjärvi found that, in the dark, the first component of the electron transport chain, plastoquinone, is more oxidized in kleptoplasts (and therefore can accept more electrons) than in the chloroplasts of the green algae. This allows photosystem II to transfer extra electrons to plastoquinone, rather than to oxygen, when it absorbs energy from light, and to avoid the formation of harmful ROS. Havurinne and Tyystjärvi also detected higher levels of non-photochemical quenching – a process in which excess excitation energy is dissipated as heat – in kleptoplasts exposed to light. These two mechanisms help to increase the longevity of kleptoplasts by dissipating excessive energy and suppressing the formation of ROS (Figure 1).

Additionally, P700 is also maintained in an oxidized state (so it can keep receiving electrons) both in *E. timida* and *A. acetabulum*. This protects the photosynthetic apparatus from light-induced damage, which improves the longevity of the kleptoplasts. In order to remain oxidized, P700 must donate excess electrons to alternative molecules. Havurinne and Tyystjärvi propose that proteins called flavodiirons may be accepting excess electrons from photosystem I, and transferring these electrons to oxygen molecules without producing ROS. However, further studies are required to confirm whether flavodiiron proteins are important for protecting the kleptoplasts of sea slugs.

It is thought that chloroplasts originated from a formerly free-living cyanobacterium being engulfed by a single-celled eukaryote more than 1.5 billion years ago (Archibald, 2015). The relationship was cemented by the transfer of part of the DNA from the engulfed cyanobacterium to the nucleus of its host over the course of evolution. Understanding how photosynthetic sea slugs are able to incorporate and maintain functional chloroplasts isolated from the remaining algal components, could provide useful insights into how photosynthetic eukaryotes evolved.
